# Resveratrol post-treatment protects against neonatal brain injury after hypoxia-ischemia

**DOI:** 10.18632/oncotarget.13018

**Published:** 2016-11-02

**Authors:** Shulin Pan, Songlin Li, Yingying Hu, Hao Zhang, Yanlong Liu, Huai Jiang, Mingchu Fang, Zhengmao Li, Kebin Xu, Hongyu Zhang, Zhenlang Lin, Jian Xiao

**Affiliations:** ^1^ Department of Neonatology, The Second Affiliated Hospital and Yuying Children's Hospital, Wenzhou Medical University, Wenzhou 325000, China; ^2^ The Institute of Life Sciences, Wenzhou University, Wenzhou 325035, China; ^3^ The School of Pharmacy, Wenzhou Medical University, Wenzhou 325035, China

**Keywords:** resveratrol, hypoxia-ischemia, brain damage, inflammation, apoptosis

## Abstract

Neonatal hypoxic-ischemic brain injury is a devastating disease with limited treatment options. Preventive treatment with resveratrol has indicated to be well tolerated and has lower toxicity in both experimental models and human patients. However, whether resveratrol administration post-hypoxic-ischemic protects against neonatal hypoxic-ischemic injury is not known. Here we reported that post-treatment with resveratrol significantly reduced brain damage at 7-day after the injury. We found that resveratrol reduced the expression levels of key inflammatory factors at the mRNA and protein levels, and at least partially via inhibiting microglia activation. Moreover, resveratrol exerted an anti-apoptotic effect, as assessed by TUNEL staining, and altered the expression of the apoptosis-related genes Bax, Bcl-2 and caspase3. Our data indicate that post-treatment with resveratrol protects against neonatal hypoxic-ischemic brain injury and suggest a promising therapeutic strategy to this disease.

## INTRODUCTION

Neonatal encephalopathy due to perinatal hypoxia-ischemia (HI) is a devastating disease that is a main cause of mortality in infants. The disease can lead to long-term neurological deficits, such as cerebral palsy, epilepsy, learning impairment and mental retardation. Currently, the therapeutic strategies for attenuating the injury in the clinical setting are limited. Hypothermia is now established as the standard treatment, but that modality is only partially effective and has a narrow therapeutic window of 6 hours [1]. Thus, finding new treatments to provide safe and efficient protection to neonates suffering from HI is critical.

Inflammation plays a critical role in mediating brain injury induced by neonatal hypoxic-ischemic brain injury. Hypoxia-ischemia triggers inflammation minutes after the insult [2], and increasing evidence shown that post-HI is responsible for the exacerbation of the brain damage. The key components of inflammation include immune cells, adhesion molecules, cytokines, chemokines and oxidative stress [3]. Another event, apoptosis, frequently occurs in pathophysiological settings of nervous system injury and disease, notably following neonatal HI [4]. Apoptosis is an important mechanism of cell death after HI in the immature brain [5] that accounts for the delayed cell death that contributes to a significant proportion of the final cell loss after HI [1, 3, 6]. Components of the apoptotic pathways are several times more pronounced in immature than in juvenile and adult brains [7]. Other mechanisms of HI-induced brain damage include oxidative stress, excitotoxicity and the activation of other cell death pathways. Because the exacerbation of the brain injury comes from various pathological processes, therapeutic strategies that can target multiple mechanisms could be useful in limiting post-HI in neonatal brain damage.

Resveratrol (3,4′,5- trihydroxystilbene; RSV), a naturally occurring polyphenolic compound, has broad physiological and pharmacological properties, including anti-aging, anti-carcinogenic, anti-inflammatory, anti-oxidant and anti-apoptotic properties [8–10]. Studies have identified a neuroprotective role for resveratrol in animal models of central nervous system diseases such as Alzheimer's disease, and cerebral ischemia/reperfusion injury [9, 11–15]. The experimental studies *in vivo* shown that resveratrol is well tolerated and has low toxicity [16, 17]. Thus the rapid adoption of resveratrol has been tested in clinical trials, including for overweight/obesity, diabetic/metabolic syndrome, cancer and cardiovascular disease [18–21]. In addition, several studies have investigated its effect on the central nervous system in human adults [22, 23]. And no adverse effects of resveratrol were reported in the majority of human studies [24]. The characteristics of being well-tolerated and low toxicity are important criteria for the application of a drug in medicine, particularly in neonates. Pretreatment with resveratrol has been reported to attenuate perinatal hypoxic-ischemic brain injury [24–26]. However, few studies have reported whether post-treatment with resveratrol can protect against neonatal hypoxic-ischemic brain injury.

In this study, we found that post-treatment with resveratrol has a neuroprotective effect on neonatal hypoxic-ischemic brain injury and that the effect is related to the inhibition of inflammation and apoptosis. The post-insult administration regimen of resveratrol has greater clinical relevance than pre-insult administration does, and its various mechanisms of action described may provide useful insights into limiting post-HI in neonatal brain damage.

## RESULTS

### RSV reduces brain tissue loss after HI injury in the neonatal rats

**Figure 1:**
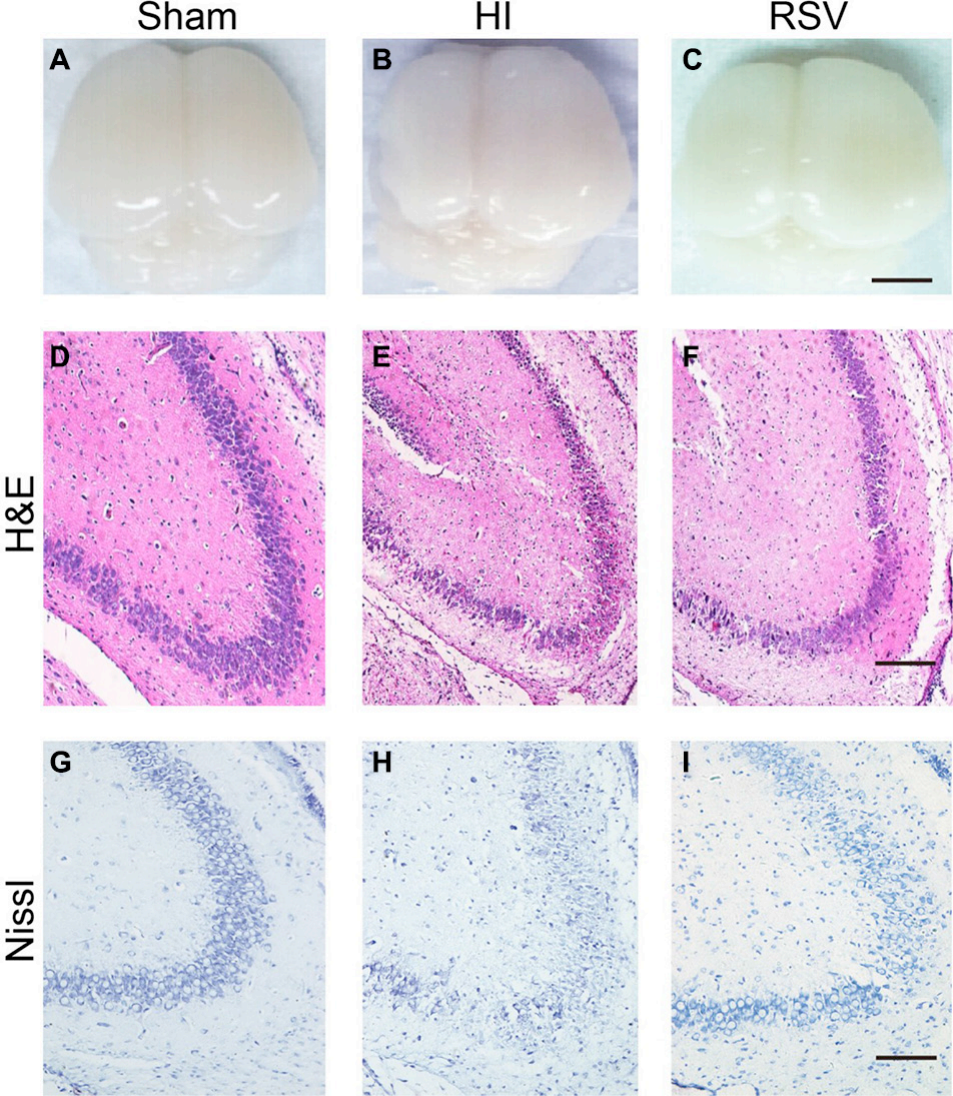
General morphology of the brain and hippocampus after hypoxic-ischemic injury (**A**–**C**) General observation of rat brains 7-day after hypoxic-ischemia from the (A) Sham, (B) HI and (C) RSV. Scale bar = 1 cm. (**D**–**F**) Representative photomicrographs of hematoxylin and eosin (H&E) stained hippocampal dentate gyrus from (D) Sham, (E) HI and (F) RSV groups at 7 days after hypoxic-ischemia. Scale bar = 200 μm. (**G**–**I**) Representative microphotoimages of Nissl stained dentate gyrus from (G) Sham, (H) HI and (I) RSV rats at 7-day after the HI injury. Scale bar = 100 μm.

To gain general information of the hypoxic-ischemic brains, the H&E staining and the MBP/MAP-2 immunohistochemical reactivity were performed using tissues collected at 7d post-injury. Brains from the sham group appeared normal; both cerebral hemispheres had a similar appearance (Figure 1A). The hemispheres ipsilateral to the ligated side from the HI group exhibited atrophy and liquefaction with an obvious collapse (Figure 1B), but the brain atrophy was alleviated in the RSV group (Figure 1C). We further compared histopathological and neuronal alterations in the cortex and hippocampal dentate gyrus after the HI injury via HE staining and Nissl staining.

**Figure 2:**
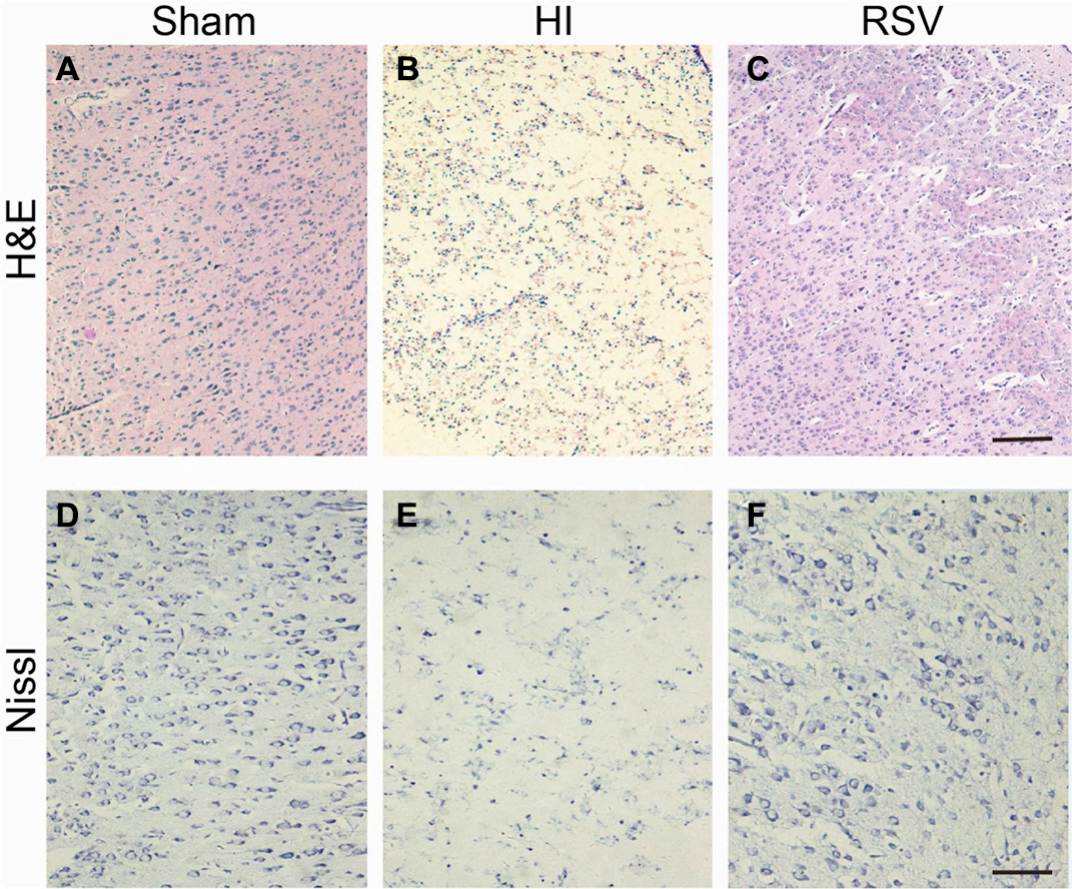
General morphology of the cortex after hypoxic-ischemia (**A**–**C**) Representative microphotographs of hematoxylin and eosin (H&E) stained cortex from (A) Sham, (B) HI and (C) RSV at 7-day after hypoxic-ischemic insult. Scale bar = 200 μm. (**D**–**F**) Representative photomicrographs of Nissl stained cortex from (D) Sham, (E) HI and (F) RSV animals at 7-day after the injury. Scale bar = 100 μm.

There was a remarkable tissue loss in the hippocampus and cortex after HI injury (Figure 1E, 1H and Figure 2B, 2E). However, this damaged brain tissue was significantly decreased in the RSV group compared to the HI group (Figure 1F, 1I and Figure 2C, 2F). Morphologically neurons with round and pale stained nuclei were normally seen throughout the cortex and hippocampus in the sham group (Figure 1D, 1G and Figure 2A, 2D). In the HI group, there was a marked increase in the number of dying cells, characterized by shrunken cells with pyknotic nuclei and even the absence of neurons (Figure 1H and Figure 2E). Post-treatment with resveratrol decreased the number of dying cells as well as the area of tissue damage (Figure 1I and Figure 2F).

**Figure 3:**
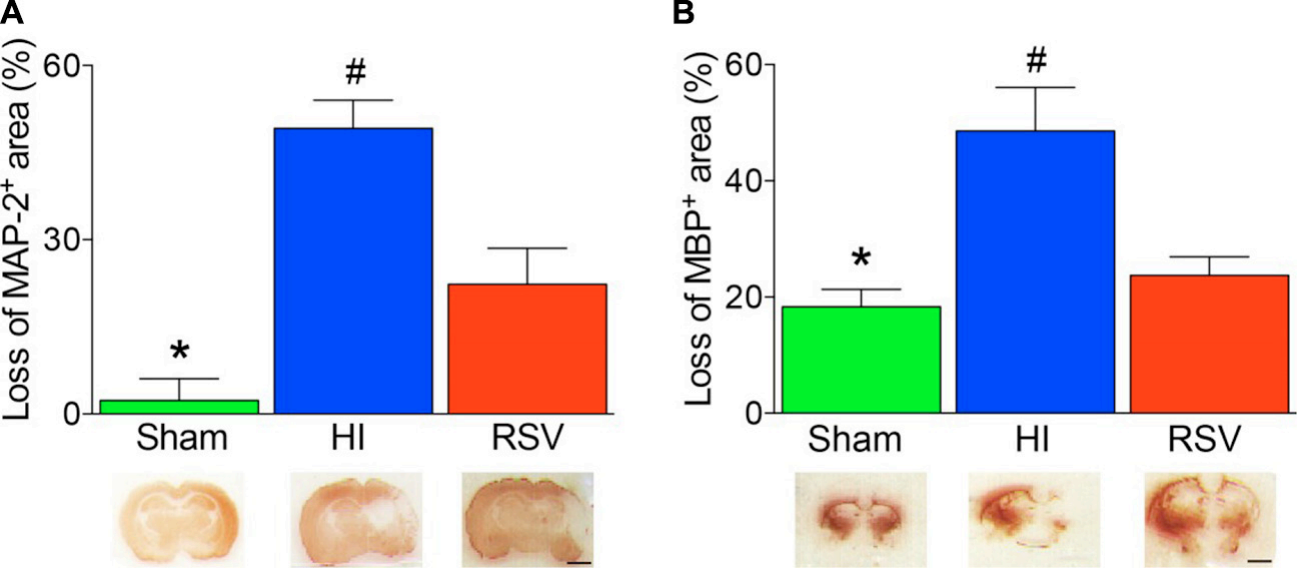
Lesion size with MAP-2 and MBP staining after hypoxic-ischemic injury The loss of MAP-2 (**A**) and MBP (**B**) areas were significantly higher in the HI group than in the groups of Sham or RSV. Representative examples of coronal sections at 7 day after hypoxic-ischemia were shown in the lower panels accordingly. Values are mean ± SEM, *n* = 4 animals per group. Sham vs. RSV, *p < 0.05; HI vs. Sham or RSV, ^#^*p* < 0.05.

In addition, there was a significant difference in neuronal (MAP2 - positive area) and oligodendrocyte (MBP - positive area) loss between RSV-treated rats and vehicle at 7 days after HI. A significant reduction in the MAP-2-positive and MBP-positive area losses was also observed in the RSV group versus the HI group (Figure 3A, 3B).

### Anti-inflammatory effects of RSV

**Figure 4:**
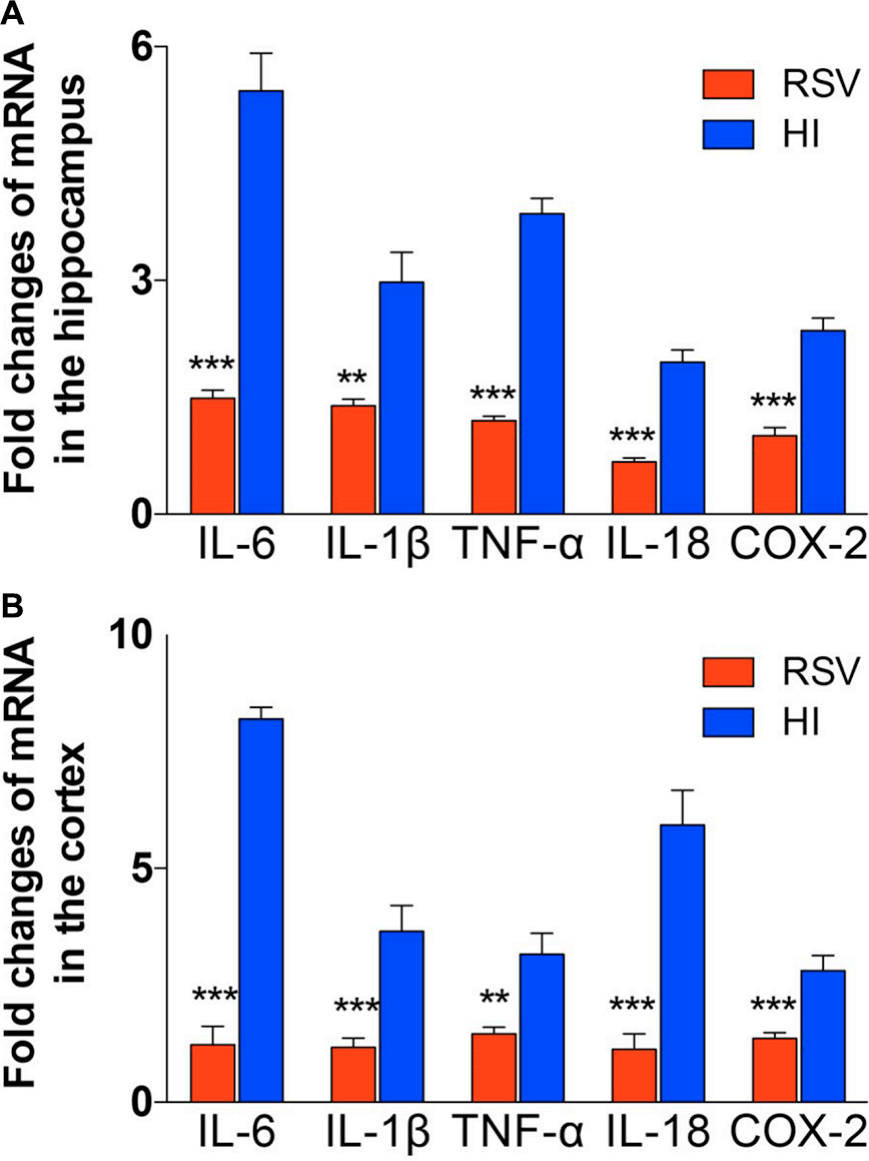
The exprestsion of inflammatory response genes in hippocampus and cortex The levels of mRNA expression in hippocampus (**A**) and cortex (**B**) 24 h after hypoxic-ischemic injury are reported as the value normalized to β-Actin for each sample. Values are mean ± SEM, *n* = 5 animals per group. HI vs. RSV, **p < 0.01, ***p < 0.001.

To test the effect of inflammation after hypoxic-ischemic brain injury, several inflammatory markers were measured 24 h after HI injury, at which inflammatory response can be reliably detected but irreversible brain damage has not yet occurred. Real-time RT-PCR was used to evaluate the effect of post-treatment with resveratrol after hypoxia-ischemia on the mRNA expression levels of various inflammatory mediators. Four cytokines (TNF-α, IL-18, IL-1β and IL-6) and another inflammatory factor COX-2 were significantly up-regulated in the hippocampus (Figure 4A) and cortex (Figure 4B) in the HI group. However, the inflammatory factors significantly decreased in the RSV group (Figure 4A, 4B).

**Figure 5:**
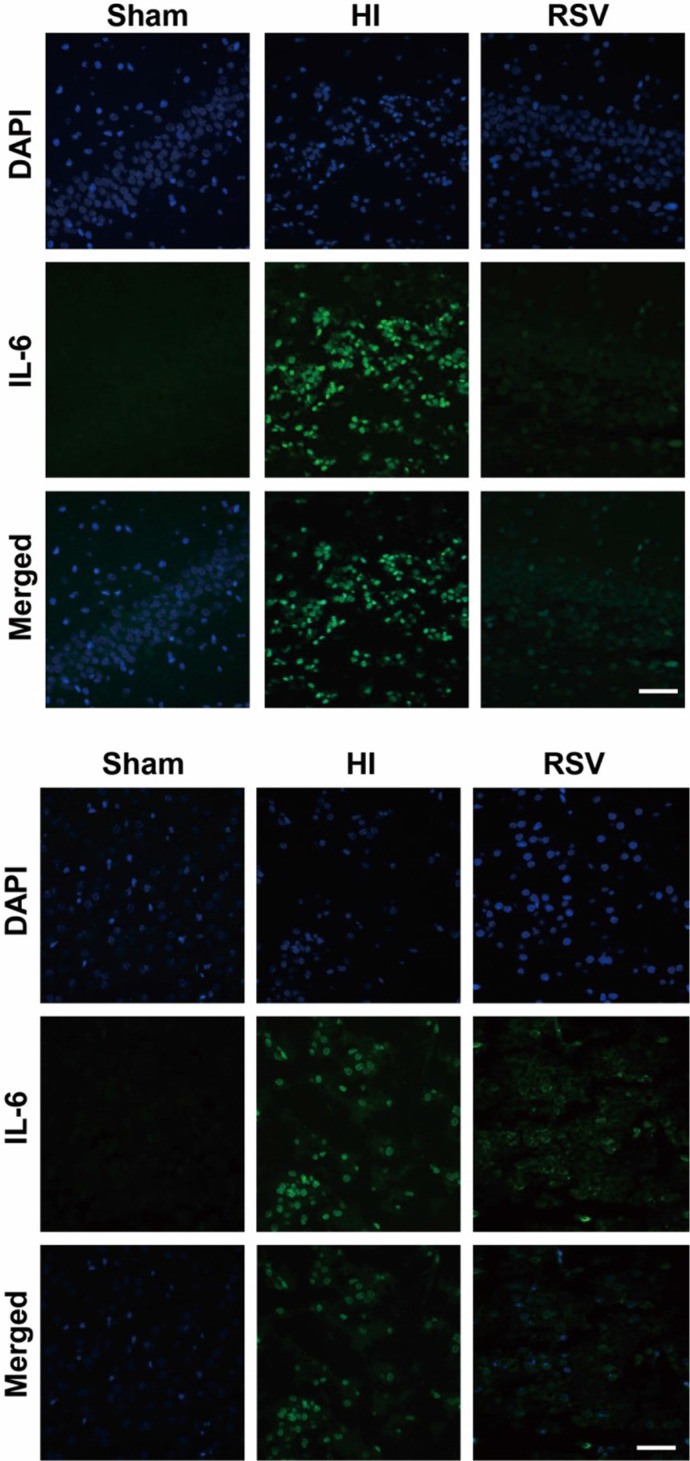
IL-6 immunohistochemistry Representative examples of microphotographs from 7 days after hypoxic-ischemia in the hippocampal dentate gyrus (**A**) and cortex (**B**). Note that the IL-6 immunoreactive signals were significantly increased in the HI group compared to the Sham, and decreased after post-treatment with RSV. Scale = 100 μm.

**Figure 6:**
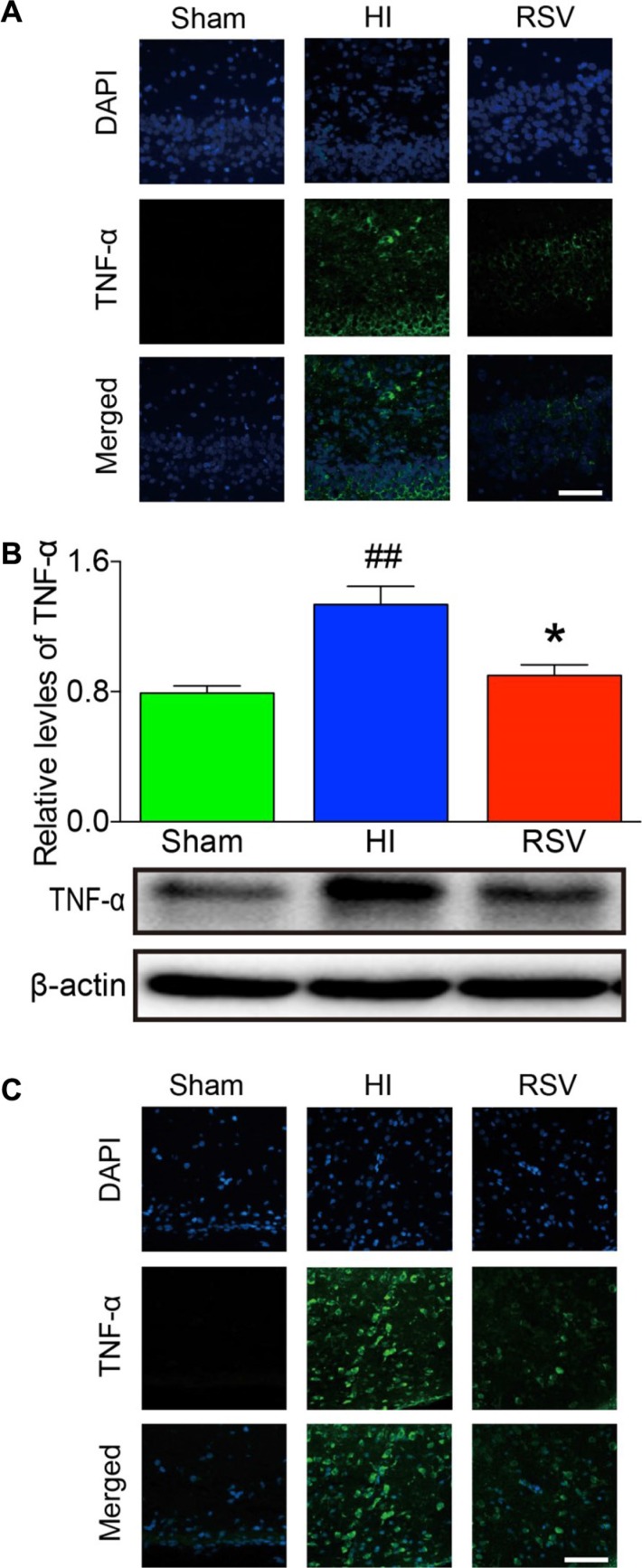
TNF-α immunohistochemistry and Western blots Representative examples of microphotographs from 7 days after hypoxic-ischemia in the hippocampal dentate gyrus (**A**) and cortex (**C**). (**B**) Western blot analysis of TNF-α levels in the hippocampus. β-Actin served as a protein loading control. The protein TNF-α contents were significantly lower in hippocampus of the RSV animals 7 days after hypoxic-ischemia than the HI animals (upper panel B). Representative Western blot detected with anti-TNF-α antibody (lower panel B). Scale = 100 μm. Values are mean ± SEM, *n* = 5 rats per group. RSV vs. HI, *p < 0.05; Sham vs. HI, ^##^*p* < 0.01.

Immunofluorescence of IL-6 and TNF-α showed a slight expression in both hippocampus and cortex in the sham group (Figure 5A, 5B and Figure 6A, 6C). Immunohistochemically, strong positive immunoreactivity for IL-6 and TNF-α were found in the HI group, suggesting a dramatic release of inflammatory cytokines in the hippocampus (Figure 5A and Figure 6A) and cortex (Figure 5B and Figure 6C). In the RSV group, we observed reduced fluorescence signal for IL-6 and TNF-α, respectively. The TNF-α western blot analysis results corresponded with the immunofluorescence of the same marker. The levels of TNF-α in the hippocampus was expressed at the lowest level in the sham group, and dramatically increased after HI (Figure 6B). The protein expression of TNF-α in the RSV group was significantly suppressed (*P* < 0.05) compared to the HI group (Figure 6B).

**Figure 7:**
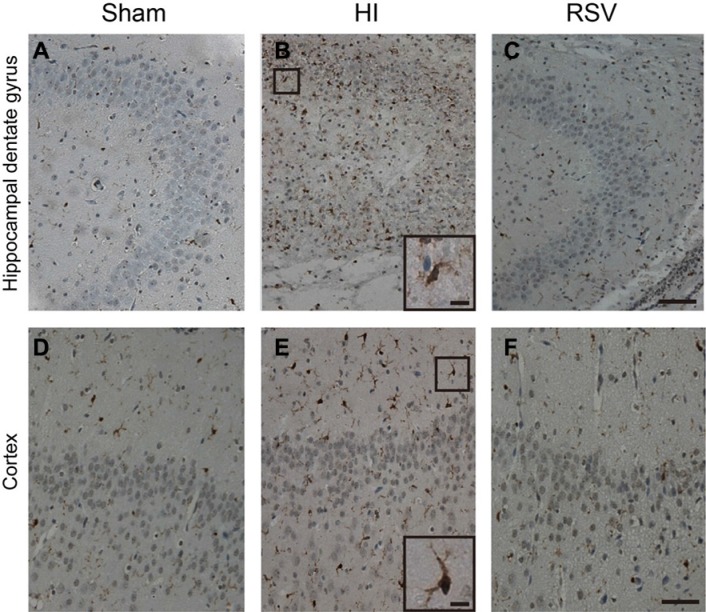
Iba-1 immunohistochemistry Representative examples of photomicrographs from 7 day after hypoxic-ischemia in the hippocampal dentate gyrus (**B**) and cortex (**E**). Note that the Iba-1 immunoreactive signals were significantly increased in the HI (**B**, **E**) group compared to the Sham (**A**, **D**), but decreased after post-treatment with RSV (**C**, **F**). Insets: Higher magnification of Iba-1 positive cells from dentate gyrus and cortex in the HI group. Scale = 100 μm (**A**–**F**) and 25 μm (insets).

Microglia are the resident inflammatory cells in the central nervous system and a prominent source of inflammatory cytokines after brain injury. We then examined the effect of RSV on microglial activation by assessing immunohistochemical staining of Iba1, a marker of microglia. The microglial numbers were enormously elevated in the hippocampal dentate gyrus and cortex after HI (Figure 7B, 7E and 7A, 7D). In contrast, the number of microglia was markedly reduced in the RSV group (Figure 7C, 7F)

### Anti-apoptotic effect of RSV

**Figure 8:**
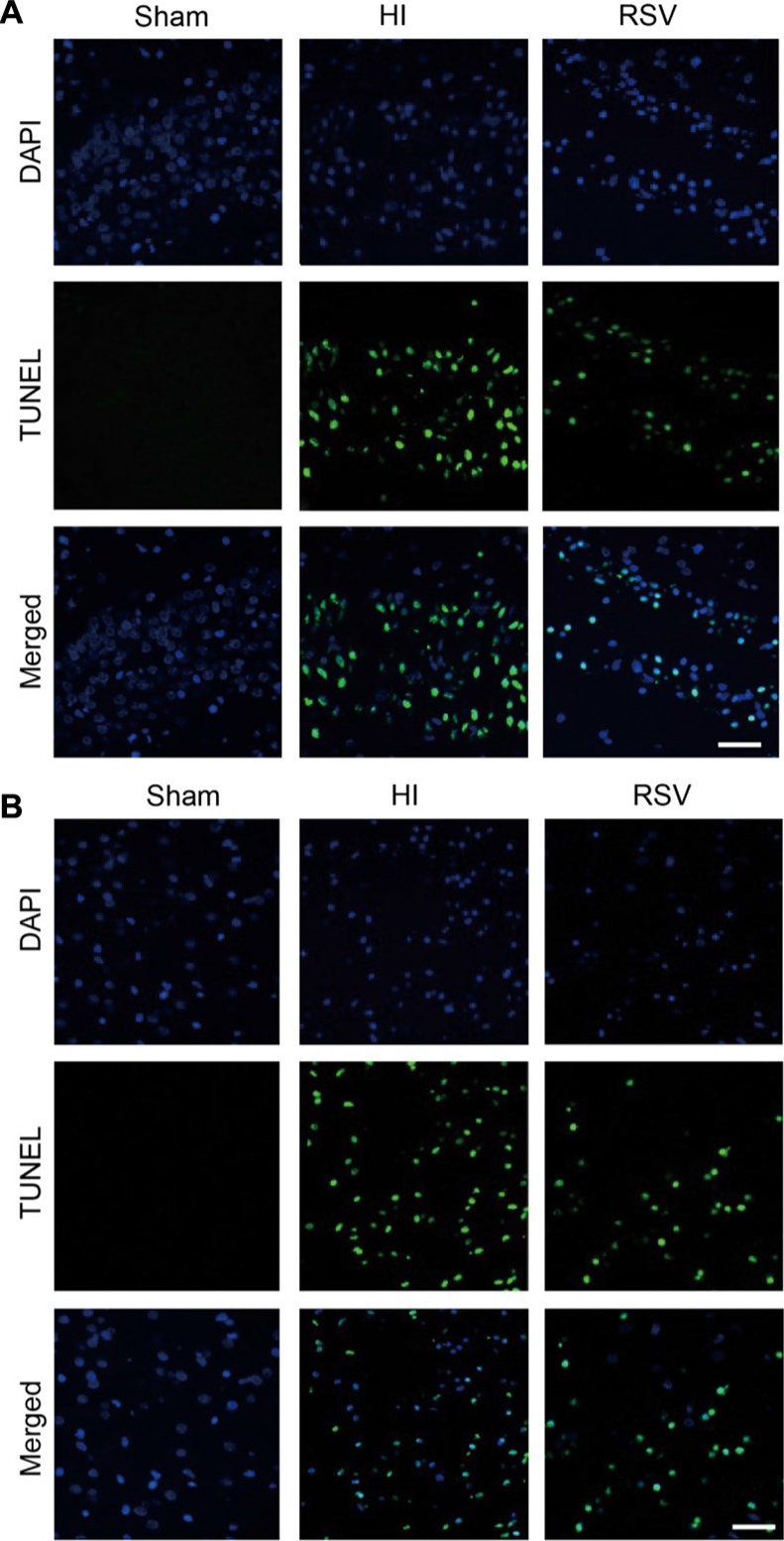
TUNEL immunohistochemistry Representative examples of microphotographs from 7 day after hypoxic-ischemia in the hippocampal dentate gyrus (**A**) and cortex (**B**). Note that the TUNEL immunoreactive signals were significantly increased in the HI group versus the Sham, and decreased after post-treatment with RSV. Scale = 100 μm.

**Figure 9:**
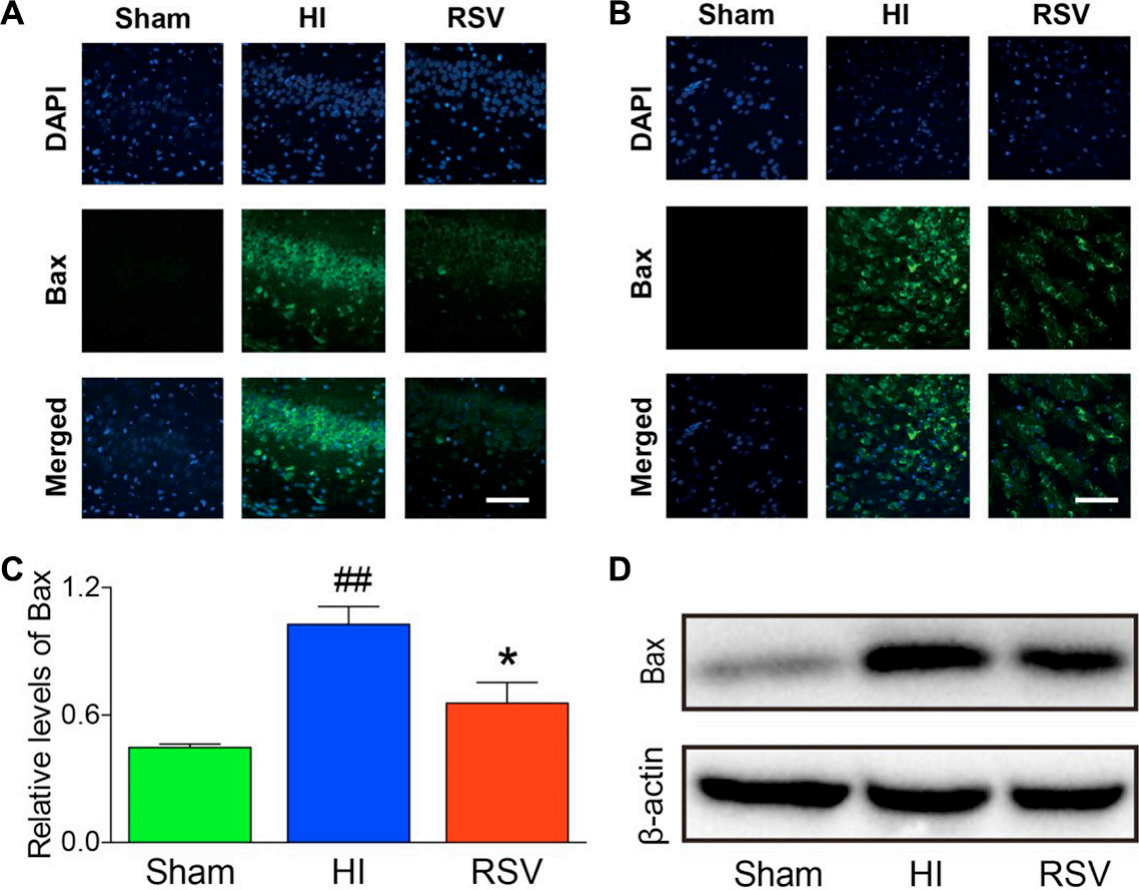
Bax immunohistochemistry and Western blots Representative examples of microphotographs from 7 days after hypoxic-ischemia in the hippocampus (**A**) and cortex (**B**). (**C**) Western blot analysis of Bax levels in the hippocampus. β-Actin served as a protein loading control. The protein Bax contents were significantly lower in hippocampus of the RSV animals 7 days after hypoxic-ischemia than the HI animals. (**D**) Representative Western blot detected with anti-Bax antibody. Scale = 100 μm. Values are mean ± SEM, *n* = 5 rats per group. RSV vs. HI, *p < 0.05; Sham vs. HI, ^##^*p* < 0.01.

**Figure 10:**
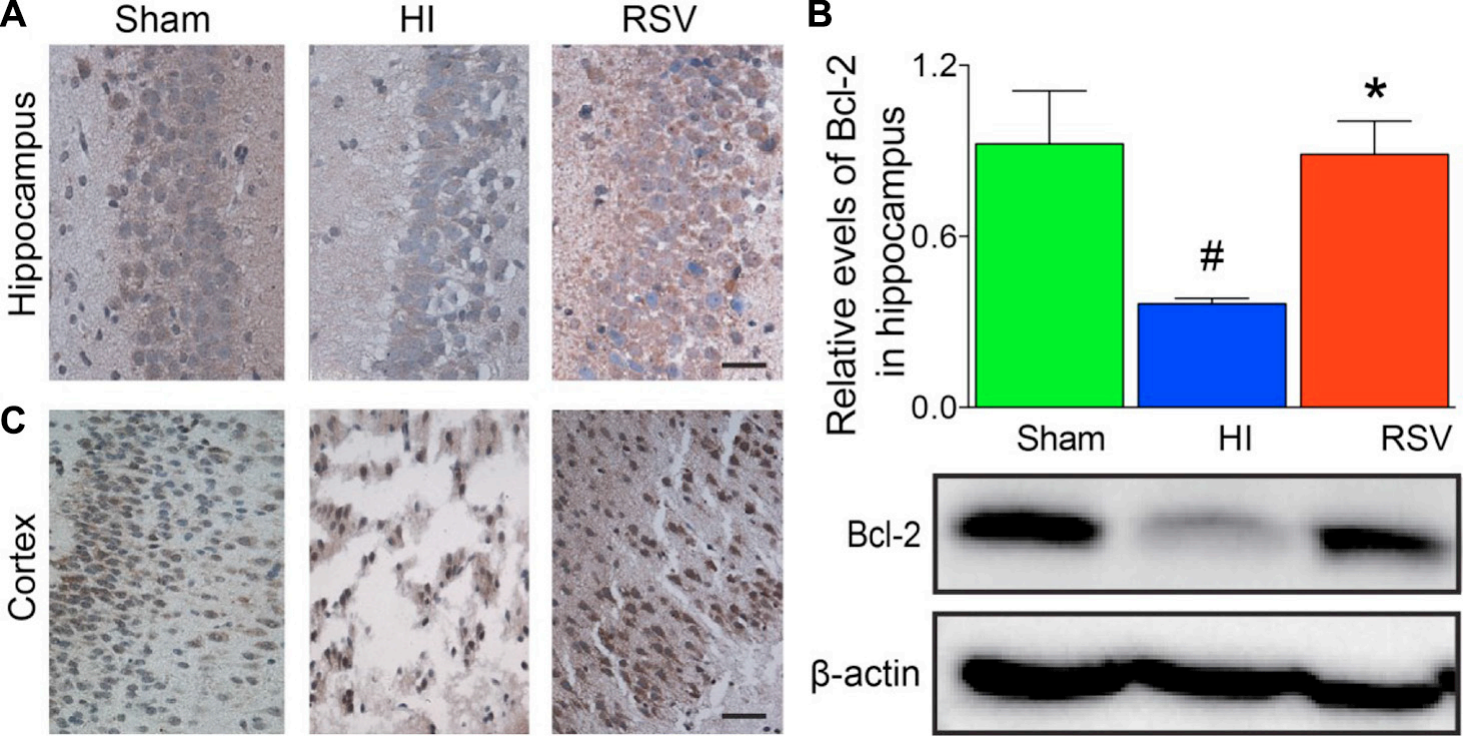
Bcl-2 immunohistochemistry and Western blots Representative examples of microphotographs from 24 hours after hypoxic-ischemia in the hippocampal CA3 (**A**) and cortex (**C)**. (**B**) Western blot analysis of Bcl-2 levels in the hippocampus. β-Actin served as a protein loading control. The protein Bcl-2 contents were significantly higher in hippocampus of the RSV animals 24 hours after hypoxic-ischemia than the HI animals (upper panel B). Representative Western blot detected with anti-Bcl-2 antibody (lower panel B). Scale = 50 μm. Values are mean ± SEM, *n* = 5 rats per group. RSV vs. HI, *p < 0.05; Sham vs. HI, ^#^*p* < 0.05.

TUNEL staining provided us with knowledge about the neuronal damage based on DNA breaks. In the hippocampus (Figure 8A) and cortex (Figure 8B) at 24 h after HI brain injury, the number of TUNEL-positive cells increased significantly in the HI group compared to the sham group. However, this increase in the number of apoptotic cells was significantly attenuated in the RSV group in comparing to the HI group (data not shown). We further assessed expression levels of several apoptosis-related genes Bax, Bcl-2 and cleaved caspase 3. Western blot, immunochemistry and immunofluorescence for the pro-apoptotic factor Bax and anti-apoptotic factor Bcl-2 revealed a consistent effect of resveratrol intervention with our data above. Resveratrol intervention obviously inhibited the HI-induced up-regulation of Bax in the hippocampus and cortex (Figure 9A–9D). The Bcl-2 levels in the brain were low in the HI group (Figure 10), whereas post-treatment with resveratrol increased significantly Bcl-2 expression after HI (Figure 10) compared with the HI group. This effect of resveratrol corresponded to its anti-apoptotic role, which was demonstrated by the reduction of TUNEL-positive cells and level of Bax (Figure 8).

**Figure 11:**
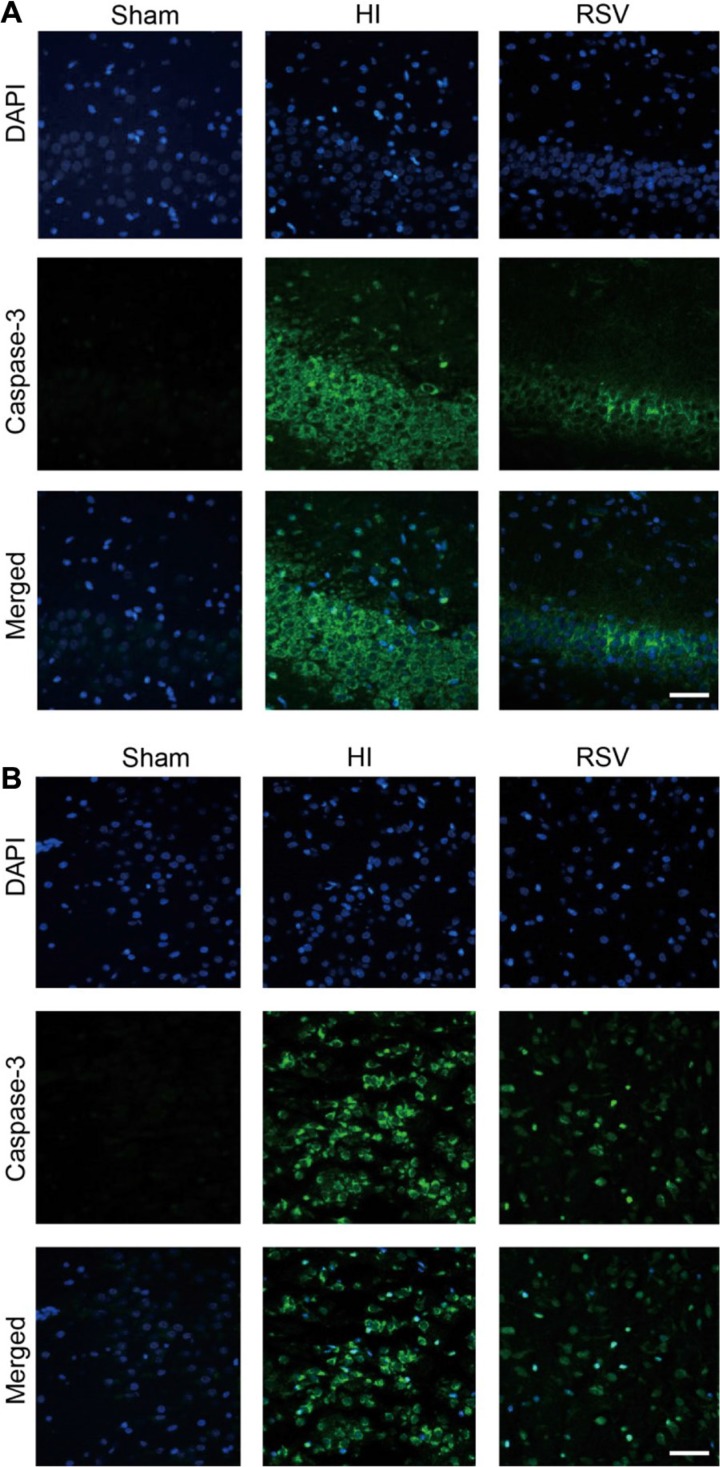
Caspase-3 immunohistochemistry Representative examples of microphotographs from 24 hours after hypoxic-ischemia in the hippocampal dentate gyrus (**A**) and cortex (**B**). Note that the Caspase-3 immunoreactive signals were significantly increased in the HI group compared to the Sham, and decreased after the RSV post-treatment. Scale = 100 μm.versus the Sham, and decreased after post-treatment with RSV. Scale = 100 μm.

Cleaved caspase 3, which is the terminal executing enzyme for cleavage of substrate, was measured 24 h after HI to examine the effects of HI and RSV on neuronal apoptosis. Immunofluorescent staining in the hippocampus (Figure 11A) and cortex (Figure 11B) revealed more caspase 3 signals in the HI group when compared to the sham. However, cleaved caspase 3-positive cells were reduced in the RSV group versus the HI group. These results indicated that the neural protective effect of resveratrol might be at least partially through modulating the expression of apoptosis-related proteins.

## DISCUSSION

Neonatal hypoxic-ischemic brain injury is an important neurological disorder associated with neonatal death and long-term disability. Its mechanisms include apoptosis, excitoxicity, inflammation and oxidative stress [3]. As the standard of clinical treatment, however, hypothermia has limited utility [27]. Here we found that post-treatment with resveratrol significantly reduced brain damage, decreased the protein levels of key inflammatory factors, and altered the expression of the apoptosis-related genes after the HI injury. Our data suggest a promising therapeutic strategy to the neonatal hypoxic-ischemic brain injury.

Resveratrol has been shown to have a number of physiological properties, such as anti-oxidant, anti-inflammatory, anti-aging and anti-viral activities. Further, the preventive administration of resveratrol has been demonstrated to play neuroprotective role in the CNS experimental models, including Alzheimer disease, Parkinson's-like disease, Huntington's disease, subarachnoid hemorrhage, traumatic brain injury and cerebral ischemia/reperfusion injury [9, 11–15]. Animal safety studies, including skin and eye irritation, dermal sensitization, subchronic, oncogenicity, reproductive toxicity and genotoxicity, were also conducted with resveratrol [16, 28], and no adverse event or reproductive toxicity were observed after the trans-resveratrol treatment in animal models [29, 30]. In the present study we failed to find any adverse effects of resveratrol on normal rats. The characteristics of being well-tolerated and low toxicity characteristics have led resveratrol to be applied in many recent clinical trials [20–22]. Furthermore, several studies have investigated the drug's effects on the central nervous system in human adults [23, 31]. Potential adverse effects of resveratrol treatment have also been tested in humans, and no adverse events have been reported in the majority of the studies [24, 55, 58]. Therefore, we believe that resveratrol could be safe for neonatal hypoxic-ischemic brain injury. To the best of our knowledge, only a couple of studies [24, 25, 55, 58] have ever reported a neuroprotective role for resveratrol in a model of neonatal hypoxic-ischemic brain injury, which showed that pre-treatment with resveratrol reduced brain damage, leading us to realize the preventative power of resveratrol. The neuroprotective studies of post-treatment with resveratrol were performed in acute brain injury models, and the other agents were found neuroprotective in the neonatal rat after hypoxic-ischemic injury [32–37]. A recently comprehensive study indicated a neuroprotection in adult brain of nonhuman primates after a long-term of resveratrol supplementary [38]. Thus, we further characterized the underlying mechanisms of the protective effect of post-treatment with resveratrol.

The general observations in the present study indicate that resveratrol may improve the brain's recovery at multiple levels. Further, Nissl and MAP-2 staining were used to identify whether neurons underwent pathological changes, and the results suggest that neuronal structure and function was maintained after administration with resveratrol. MBP staining revealed the protective effect of resveratrol on white matter. The apparent tissue loss and neuronal death at 7 days in the HI group indicate that the immature brain is unable to repair itself from this injury; however, resveratrol may preserve the structure and morphology of injured tissues and prevent dysfunction in the affected neurons. In contrast to Arteaga1 et al. [25], we found that increasing both the dosage and the treatment duration of resveratrol could improve its effectiveness.

The accumulated evidence indicates that neonatal hypoxic-ischemic brain injury is progressive and that the resulting neuropathies are linked to the activation of neuroinflammatory processes. Resveratrol has been reported to have an anti-inflammatory function and to improve the outcome of several diseases [39]. Our studies found that the inflammatory effect does contribute to the model of neonatal hypoxic-ischemic brain injury. The inflammatory response after injury is characterized by increased expression of inflammatory factors at both the RNA and protein levels [40–45]. Our experiments identified the significant expression of these inflammatory factors in the hippocampus and cortex, and further demonstrated that resveratrol plays a protective role in the inflammatory cascade by inhibiting their mRNA expression and protein production. Cytokines in the CNS are primarily derived from immune cells. Microglia are a major component of the immune cells in the CNS and strongly activated in the neonatal brain [3, 44, 46, 47]. When an ischemic event occurs, microglia are activated, and they release inflammatory factors leading to the deterioration of the injury. We did find that the IBA-1-like immunoreactive signal was deceased following the resveratrol, indicating that resveratrol attenuated the activation of microglia, and suggesting that the decreased inflammatory cytokines partly derives from suppression of activated microglia. However, considering M1/M2 phenotypes switching and balance [48–50], it would be interesting to further determine the microglia/macrophage M1 and/or M2 phenotypes in the hypoxic-ischemia brain.

Apoptosis is another critical event in hypoxic-ischemic brain injury. Previous studies shown that many apoptosis-related factors, such as caspase-3, Apaf-1, Bcl-2 and Bax, are normally expressed at a high level in the immature brain [7], showing that apoptosis is more prominent in the immature brain than the adult [51, 52]. Resveratrol has been reported to have an anti-apoptotic activity [10, 53], We asked whether an anti-apoptotic effect contributes to the protective role of post-treatment with resveratrol in neonatal HI. Bax-dependent mitochondrial outer membrane permeabilization is a critical event in the apoptotic mechanism that occurs in the immature brain [25, 46, 54]. After HI, Bax translocates from the cytosol to the mitochondrial outer membrane and forms pores, leading to mitochondrial permeabilization followed by activation of apoptotic cell death [46]. Bax-inhibiting peptide has a neuroprotective effect on neonatal hypoxic-ischemic brain injury [54], but not the corresponding injury in adult [47], showing the crucial role of Bax in the apoptotic mechanism after neonatal hypoxic-ischemic brain injury. Caspase-3 is a critical executioner of apoptosis. The caspase-dependent pathway for the induction of apoptosis converges upon the activation of caspase-3. The extent of caspase-3 cleavage and activation following brain injury is greater in developing rodents compared to adults [4]. Therefore, Bax and caspase-3 have become the main focus in apoptosis studies. In our experiments, DNA fragmentation, the expression of Bax and cleaved caspase 3 were inhibited by resveratrol, suggesting that resveratrol suppresses the initiation of apoptosis to some extent. Bcl-2, which has been reported to be involved in mechanisms other than those based on direct interaction with Bax, was elevated by resveratrol leading to an anti-apoptotic effect. The anti-apoptotic effect of resveratrol may contribute to the recovery of brain tissue and neuronal function.

Overall, to evaluate the potential neuroprotection of resveratrol after neonatal hypoxic-ischemic brain injury, we examined the histological changes in the brain. We observed that resveratrol promotes the recovery of tissue loss and has an anti-inflammatory role by inhibiting the injury-induced up-regulation of inflammatory factors at the mRNA and protein levels. Further, we found that resveratrol decreases DNA fragmentation, the protein expression of Bax and caspase 3 cleavage and increases Bcl-2 expression. The decreased tissue loss might be due to the protective role of resveratrol in inhibiting inflammation and apoptosis during the acute interval after injury. Thus we identified the neuroprotective role for post-treatment with resveratrol against the neonatal hypoxic-ischemic brain injury. We also found that the protective role and potential therapeutic value of resveratrol for treating neonatal HI brain injury was associated with anti-inflammatory and anti-apoptotic mechanisms. It is noted that current study was carried out with one single dose of resveratrol application. Since neonatal hypoxic-ischemic brain injury is a complicated injury, and targeting only one mechanism is far from sufficient, exploring the timing, dosage and routes of the drug administration, the relationship between apoptosis and inflammation, and the functional studies should be addressed in the future.

## MATERIALS AND METHODS

### Rat model of HI injury and RSV administration

All animal procedures were performed in accordance with the Animal Center of the Chinese Academy of Science (Shanghai, China) and the Laboratory Animal Ethics Committee of Wenzhou Medical University. Efforts were made to minimize animal suffering and to minimize the number of animals used. Pregnant rats (Sprague-Dawley) were purchased from the Animal Center of the Chinese Academy of Sciences and were housed individually, maintained on an *ad libitum* feeding schedule, and kept on a 12-h light/dark cycle. The hypoxia-ischemia (HI) model was produced on postnatal day 7 (P7) rat litters as described [55] with modification. Briefly, pups were anesthetized with 3% isoflurane and the balance of room air, and the left common carotid artery was ligated. The rats were sutured, 1.5 h for recovery, and placed in a glass chamber containing a humidified atmosphere of 8% oxygen/92% nitrogen, which was submerged in a 37.5°C water bath to maintain normothermia. Pups were kept in hypoxic chambers for 2.5 h and then returned to their dam. A sham group that had a ligature placed in an identical fashion without actually occluding the vessel or without undergoing hypoxia serviced as controls. Resveratrol (Sigma Chemical Co., UK) was freshly prepared by dissolving and diluting in 2% ethanol. Resveratrol (100 mg/kg, i.p.) was injected three times at 0 h, 8 h and 18 h, respectively, after hypoxic-ischemic brain injury (groups of HI plus resveratrol) [56, 57], while the HI group received 2% ethanol alone (i.p.) and the volume of the 2% ethanol was calculated by using the following equation: (100 × the amount of all pups in the HI group × the weight of the pup in the sham group)/total weight of the pups in the HI group. These rats were sacrificed at 24 h after the injury and performed experiments for detecting message and protein levels of anti-inflammatory factors, and immunohistochemistry signal of Iba-1, Bcl-2, Bax, and caspase3, respectively. Those brains collected at 7d post-injury for general observation, H&E, and signal of MBP/MAP-2 were received two additional injections of resveratrol (100 mg/kg, i.p.) at 48 h and 72 h following the hypoxic-ischemic injury.

### Histochemistry and Immunohistochemistry

Rats were deeply anesthetized and transcardially perfused with 20 ml of saline, then with 20 ml of 4% paraformaldehyde (PFA) in 1 × phosphate buffered saline (PBS) for 5 min. Brains were removed and kept in 4% PFA for a post-fix overnight. After dehydration the brains were embedded in paraffin, and sliced coronally in 5-μm. Tissue sections were initially subjected to hematoxylin and eosin (HE) staining and Nissl staining for general assessment of histopathology.

Additional sets of sections were dewaxed in xylene overnight and subsequently passed through 3 × 10 min 99% ethanol and 2 × 10 min 96% ethanol. Endogenous peroxidase activity was then blocked with 0.35% H_2_O_2_ (35% H_2_O_2_ diluted 1:99 in methanol) for 30 min. Rehydration was completed by a rinse in 96% ethanol, 10 min in 70% ethanol and 3 rinses in distilled water, and then incubated with 5% bovine serum albumin (BSA) for 30 min at room temperature. Antibodies (Bcl-2, sc-492, 1:150, Santa Cruz Biotechnology; Iba-1, 1:400, ab5076, Abcam; MAP-2, 1:200, sc-20172, Santa Cruz Biotechnology; MBP, 1:200, sc-13914, Santa Cruz Biotechnology; TNF-α, 5 μg/ml, ab9755, Abcam; IL-6,1:200, sc-1266, Santa Cruz Biotechnology; Bax, 1:200, sc-493, Santa Cruz Biotechnology and cleaved caspase 3, 1:400, #9579, Cell Signaling Technology) were diluted in 10 mM PBS containing 0.1% BSA, 0.3% Triton X-100, respectively, and the sections were incubated with the primary antibodies overnight for 1 h at RT and subsequently at 4°C overnight. The horseradish peroxidase (HRP) conjugated secondary antibodies were incubated at room temperature for 1 h, followed by 3,3′-diaminobenzidine (DAB) staining (Bcl-2, Iba-1 MAP-2, MBP and IL-6), or incubation of the Alexa Fluor 488 conjugated secondary antibodies (TNF-α, IL-6, Bax and cleaved caspase 3). Negative controls were performed using the same procedure described above in the absence of the primary antibody. The tissues were counterstained with DAPI, mounted with Anti-fade Mounting Medium. The images of MAP-2 and MBP were captured with a scanner (imageCLASS MF4322d, Canon) and others were visualized using a Nikon ECLIPSE Ti microscope (Nikon, Tokyo, Japan). The extent of tissue damage was determined by calculating the amount of surviving tissue in each section. Briefly, brain damage was analyzed using the ImageJ software (http://imagej.nih.gov/ij/) by outlining both hemispheres on full section images. The ipsilateral MAP-2 area was calculated as a percentage for each animal using the following equation: [1 - (area ipsilateral MAP-2 staining/area contralateral MAP-2 staining)] × 100. The MBP area was determined as same as the calculation method of the MAP-2 area loss. For TUNEL (terminal deoxynucleotidyltransferase-mediated dUTP nick end labeling) staining, sections were incubated with TUNEL reaction mixture in a dark humidified chamber for 1h at 37°C, followed by a final wash for 3 × 10min with PBS. Apoptotic cells were characterized by green fluorescence of the nucleus and nuclear membrane according to the manufacturer's protocol. Quantitation was performed by counting the number of positive cells in five randomly chosen fields within each slide at 400× magnification using a Nikon ECLIPSE Ti microscope. The index of apoptosis was calculated as the ratio of the overall number of apoptotic cells to the total number of cells.

### Quantitative real-time RT- PCR

Cohorts of animals were decapitated, the brains rapidly removed and cortex dissected free on an ice block, frozen in liquid nitrogen and stored at 80-C. The tissue samples were homogenized in TRIzol reagent (TriPure Isolation Reagent, Roche Applied Science). Total RNA was extracted from the tissue according to the manufacturer's protocol. The extracted RNA was quantified by UV spectrophotometry; only those samples with a 260:280 ratio of greater than 1.8 were used. Total RNA was reverse transcribed in duplicate. A third RNA sample was incubated without reverse transcriptase (no-RT control). Totally 0.5 μg of RNA was used to synthesize the cDNA using the Prime Script™ RT reagent Kit and Bio-Rad MyCycler TM^™^ Thermal Cycler for the reverse transcription polymerase chain reaction. Samples were run in parallel with each primer set in real-time PCR with bio-radiQ^™^ SYBR^®^ Green Supermix. β-actin serviced as a housekeeping control. The forward and reverse primer sequences are shown in Table 1. The specificity of each PCR reaction was determined with melting curve analysis, which is a measure of product length and GC composition and provides a melting temperature that is specific for each amplicon [58]. To further refine the measurement of PCR amplification in this process, the amplification signal for each PCR reaction was measured at a temperature set below the melting point for the amplicon, but above any non-specific fluorescent signal in the reaction. The crossing point for the gene of interest was normalized for the efficiency of each primer set at that PCR cycle length using efficiency curves generated by serial dilution of RNA standards with each primer set, and to the crossing point for β-actin for each sample [59].

**Table 1:** Primers used in the studies

Gene	Forward primers	Reverse primers
COX-2	CGGAGGAGAAGTGGGGTTTAGGAT	TGGGAGGCACTTGCGTTGATGG
IL-18	AAACCCGCCTGTGTTCGA	TCAGTCTGGTCTGGGATTCGT
IL-1β	CACCTCTCAAGCAGAGCACAG	GGGTTCCATGGTGAAGTCAAC
IL-6	GAGTTGTGCAATGGCAATTC	ACTCCAGAAGACCAGAGCAG
TNF-α	TACTCCCAGGTTCTCTTCAAGG	GGAGGCTGACTTTCTCCTGGTA
β-Actin	AAGTCCCTCACCCTCCCAAAAG	AAGCAATGCTGTCACCTTCCC

### Western blot

Brain tissues were lysed in RIPA buffer (25 mM Tris-HCl, 150 mM NaCl, 1% Nonidet P-40, 1% sodium deoxycholate, and 0.1% sodium dodecyl sulfate) with supplemental protease and phosphatase inhibitors (GE Healthcare Biosciences, Piscataway, NJ, USA). After centrifugation, the extracts above were quantified with bicinchoninic acid (BCA) reagents (Thermo, Rockford, IL, USA). The complex was then centrifuged at 12,000 rpm, and the supernatant was obtained for the protein assay. Proteins (30 μg for TNF-α, Bcl-2, and Bax) were subjected to sodium dodecyl sulfate polyacrylamide gel electrophoresis (SDS-PAGE) using 12% gels and blotted on to nitrocellulose membranes. After blocking, membranes were incubated with primary antibody (TNF-α, 1:3000, ab9755, Abcam; Bcl-2, 1:1000, #2876, Cell Signaling; Bax, 1:200, sc-493, Santa Cruz Biotechnology). Then, horseradish peroxidase-conjugated secondary antibodies (1:3000) were incubated with the blots for 1 h at room temperature. Western blotting of β-actin (1:3000, Bioworld, AP0060) was performed to demonstrate equal protein loading, and used as an internal control. Western blots were scanned and quantified through optical density measurements by ChemiDoc XRS+ Imaging System (Bio-Rad). Each sample was analyzed in 2 or 3 times in independent experiments, and the densitometric values of the bands on the western blots acquired by the Image Lab software (BioRad).

### Statistical analysis

Student's *t* test or factorial analysis of variance with Tukey *post hoc* comparisons was performed (GraphPad Prism 5). Results are shown as the mean ± SEM. *P* < 0.05 indicated a significant difference.
